# Chemosensory Sensitivity after Coffee Consumption Is Not Static: Short-Term Effects on Gustatory and Olfactory Sensitivity

**DOI:** 10.3390/foods9040493

**Published:** 2020-04-14

**Authors:** Alexander W. Fjaeldstad, Henrique M. Fernandes

**Affiliations:** 1 Flavour Institute, Aarhus University, Palle Juul-Jensens Boulevard 99, 8200 Aarhus N, Denmark; henrique.fernandes@clin.au.dk; 2 Flavour Clinic, ENT Department, Holstebro Regional Hospital, Laegaardsvej 12, 7500 Holstebro, Denmark; 3 Center for Eudaimonia and Human Flourishing, University of Oxford, Oxford OX1 2JD, UK; 4 Center of Functionally Integrative Neuroscience, Aarhus University, Noerrebrogade 44, 1A, 8000 Aarhus, Denmark; 5 Center for Music in the Brain, Department of Clinical Medicine, Aarhus University & The Royal Academy of Music Aarhus/Aalborg, Noerrebrogade 44, 1A, 8000 Aarhus, Denmark

**Keywords:** taste, smell, gustatory sensitivity, olfactory sensitivity, coffee

## Abstract

Chemosensory sensitivity has great variation between individuals. This variation complicates the chemosensory diagnostics, as well as the creation of a meal with universally high hedonic value. To ensure accurate characterization of chemosensory function, a common rule of thumb is to avoid food/beverages one hour before chemosensory testing. However, the scientific foundation of this time of fast remains unclear. Furthermore, the role of coffee on immediate chemosensitivity is not known and may have implications for optimization of gastronomy and hedonia. The aim of this study is to investigate the modularity effects of coffee consumption on immediate gustatory and olfactory sensitivity. We included 155 participants. By applying tests for olfactory and gustatory sensitivity before and after coffee intake, we found no changes in olfactory sensitivity, but significantly altered sensitivity for some basic tastants. We repeated our experimental paradigm using decaffeinated coffee and found similar results. Our results demonstrate that coffee (regular and decaffeinated) alters the subsequent perception of taste, specifically by increasing the sensitivity to sweet and decreasing the sensitivity to bitter. Our findings provide the first evidence of how coffee impacts short-term taste sensitivity and consequently the way we sense and perceive food following coffee intake—an important insight in the context of gastronomy, as well as in chemosensory testing procedures.

## 1. Introduction

As a prudently regulated multisensory apparatus, all senses come into play during food consumption to avoid spoiled foods, ensure adequate nutritional intake, and increase the pleasure of eating. In this multisensory cascade of peripheral and central processing, the different sensory modalities are known to have a complex pattern of inhibitory and super-additive interrelations [1]. This has led to an evolving field of research, where cross-sensory interactions are being unveiled to increase enjoyment of food [2], reduction in use of potentially unhealthy ingredients such as sugar and salt [3], and unveiling potential bias when conducting sensory research [4].

The enhancement and suppression of sensory stimuli also exists within single senses, where e.g., taste-taste interactions may cause alterations in subsequent perception [5]. For more than a century, bitter tastants have been known to inhibit perception of sweet taste [6], perhaps as an evolutionary strategy to enhance bitterness signals for potential poisonous compounds. A textbook example of a such a bitter foodstuff is coffee: The orthonasal aromas lures for consumption until the bitter substance reaches the gustatory receptors. As a gatekeeper, the activation of bitter receptors initially puts a holt to any further need for consumption. However, bitter tastants that would previously have been categorically rejected for consumption after gustatory evaluation, often become accepted or even enjoyed as experience and preferences are developed [7]. This is emphasized by the overwhelming popularity of coffee; one of the most commonly consumed beverages worldwide.

As a potent activator of both olfactory and gustatory receptors, coffee is often prohibited prior to chemosensory testing for up to an hour to avoid spillover effects on subsequent olfactory [8] or gustatory sensitivity [9]. This is not without physiological reason, as coffee contains caffeine—a phosphodiesterase-inhibitor/adenosine-receptor agonist, which may alter chemosensory perception in several aspects. Coffee can augment dynamic cerebral blood flow [10], influence brain metabolism [11], and enhancement of attention and alertness [12]. Furthermore, bitter substances can directly alter sensitivity for other tastants. The bitter substance quinine in various concentrations inhibits sweet-sensitive gustatory nerves due to the direct inhibition of the TRPM5 taste receptor [13]. A similar mechanism of bitter-sweet inhibition has been found for caffeine through another taste receptor (type 3 IP3 receptor) [14].

Beyond the taste receptor level, gustatory signaling is conveyed to the brain stem, onwards through the thalamus before reaching the primary gustatory cortex and secondary areas of processing. While the simple taste reflexes occurring at the brain stem level seems to be impervious to modulatory effects [15], the higher cognitive layers involved in taste processing are more prone to multisensory modulation [1].

With over 900 volatile compounds identified in roasted coffee [16], it is a vigorous olfactory stimulant with a broad array of olfactory receptor activation. Olfactory processing is initiated by the binding of odorants to an olfactory receptor; already at this stage odors can affect subsequent processing through olfactory adaptation [17]. Already at the location of the first olfactory synapse in the olfactory bulb, modulatory effects occur [18]. However, habituation of olfactory bulb responses seems to be linked to peripheral adaptation [19]. Central olfactory adaptation is mainly assumed to arise in the piriform cortex [20]. Thought, it may be linked to modulatory signals from the primary gustatory cortex [21]. Furthermore, the known modulatory effects of caffeine on attention may interact with olfactory function through the thalamus [22]. As such, multiple potential avenues of coffee-related alterations of chemosensory sensitivity exist at different levels of flavor processing.

Currently, only a few studies have investigated the effects of coffee on subsequent chemosensory function. Although coffee has been proposed by fragrance sellers as an enhancer for perfume discrimination, it does not seem to have any superior qualities in the enhancement of olfactory discriminatory abilities [23]. Meusel et al. investigated the effects caffeine on patients with reduced olfactory function 45 min after coffee consumption, but found no effect [24]. Han et al. found that caffeinated coffee resulted in significant attentional enhancement 30 min after consumption, but found no increase in olfactory function evaluated by olfactory threshold, discrimination, and identification scores [25]. Still, no effects on chemosensory function has yet been identified after coffee consumption.

The aim of the current study is to investigate the immediate effects of coffee consumption on olfactory and gustatory sensitivity. Furthermore, the study aims to evaluate if these effects are driven by caffeine or the other flavor components of coffee.

## 2. Material and Methods

### 2.1. Participants

A total of 156 participants, aged between 18 and 39 years, were included in this study (see Table 1 for demographics). In the primary study—“Regular coffee”—101 healthy participants were included based on a power calculation using published taste test data [9] (largest tastant SD = 1.25; α = 0.05; Power = 0.8; difference to detect = 0.35). In the subsequent exploratory study—“Decaffeinated coffee”—specific tastant-related changes were re-tested using decaffeinated coffee. Here, 55 participants were included based on tastant sensitivity changes in the primary study (SD = 1.25; α = 0.05; Power = 0.8; difference to detect = 0.50). See Table 1 for demographics.

None of the participants stated to be suffering from diseases affecting the mouth, nose, and sense of taste or smell, and had a normal subjective sense of taste and smell, assessed by a questionnaire. Furthermore, all participants had normal sense of smell–assessed by the Sniffin’ Sticks olfactory test (Burghart Messtechnik GmbH, Wedel, Germany), normal measured sense of taste–assessed with the Taste-Drop-Test. PROP sensitivity was tested and weekly average coffee consumption was registered. Participants were instructed to avoid eating, drinking, chewing gum and smoking in the hour preceding chemosensory testing. Each participant was tested by the same tester, under the same conditions and at the same location.

The study was conducted according to the Declaration of Helsinki on Biomedical Research Involving Human Subjects and was approved by the regional Ethics Committee. Permission to conduct the study was given by the Danish Ethical Committee (Etisk komité, Central Denmark Region). During enrolment, subjects were given detailed information about all testing procedures. Written consent was obtained from all subjects prior to the study.

### 2.2. Study Design and Setting

In this prospective study, participants served as their own controls. Participants were tested for baseline olfactory and gustatory sensitivity in a large ventilated room without contaminating odors, visual or auditory distractions. This took approximately 35 min. During the gustatory sensitivity test procedure, participants cleansed the mouth between stimulations by drinking tap water (room temperature) that was swirled in the oral cavity. A high re-test-reliability has been established for this procedure, where subsequent testing did not reveal significant changes in detection threshold for any tastants and tap water was found to be the most neutral water type [9]. As such, the baseline gustatory sensitivity is defined as gustatory tastant detection threshold after water consumption.

After a 15-min break, participants were served a lukewarm espresso and instructed to drink it in two sips and to ensure that the coffee was swirled around the entire oral cavity. Within two minutes, participants were provided with 150 mL of tap water and instructed to swirl each sip and drink all of the water. This was done to cleanse their palates prior to retesting of olfactory threshold and gustatory sensitivity, which took approximately 20 min.

To investigate if changes in gustatory sensitivity were related to caffeine intake, we conducted an additional exploratory study using identical testing procedures before and after consumption of de-caffeinated coffee in a new group of participants. To control for differences flavor and intensity, we used the decaffeinated version of the same coffee type used earlier (i.e., same flavor and intensity). See Supplementary Figure S1 for study timeline.

### 2.3. Olfactory and Gustatory Testing

#### 2.3.1. Olfactory Testing Procedure

A validated version of the “Sniffin’ Sticks” extended olfactory test was applied at baseline and included testing of olfactory threshold, discrimination, and identification score (collectively characterized as TDI-score) [26].

As application of the entire TDI-score would result in delayed measurement since coffee exposure, the most affectable sub-score of olfactory sensitivity was chosen as a measure for change in olfactory sensitivity, namely olfactory threshold [4]. The olfactory threshold test comprises 16 triplets of felt tip pens. For the triplets, one pen is filled with 4mL of N-Butanol dissolved in propylene glycol in increasing concentrations, and the remaining two pens are odorless (pure propylene glycol). The forced-multiple-choice-test is applied by presenting the participant with increasing concentrations of N-butanol [26,27]. When the participant has successfully identified the pen with N-butanol twice for a given concentration, the turning point is noted and pens of decreasing concentrations are presented until an error is made. Then, increasing concentrations are once again presented to the participant. This process is repeated until seven turning points are completed. The olfactory threshold is defined as the mean of the last four turning points [27]. Olfactory threshold testing was repeated after coffee intake.

#### 2.3.2. Gustatory Testing Procedure

For gustatory sensitivity, the Taste-drop-test was applied to assess the recognition threshold [9]. This test consists of ten steps of tastant dilutions for sweet (sucrose), sour (citric acid), salty (NaCl), and bitter (quinine) in tap water solvent, with a halving of tastant concentration in every dilution step.

The Taste-drop-test tastants are presented to participants with a transfer pipette (approximately 20 μL) to the desired region of the tongue. One drop is placed on each side of the anterior third of the tongue to test overall anterior taste sensitivity. A list of options consisting of “neutral”, “sweet”, “sour”, “salty”, and “bitter” is then presented to the participant who has to decide on one of these taste qualities without delay. Participants are given a glass of water (room temperature), and asked to rinse between tastings. The participant has to confirm that the former taste had disappeared before the next tastant is presented.

All four tastants are presented in semi-randomized rounds with the lowest concentration presented first (dilution step 10), before initiating the next round where four tastants in the second lowest concentration (dilution step 9) are presented.

When a tastant is correctly identified, the one step lower concentration of this tastant is presented as the first tastant of the next round; if this lower concentration is not identified, the previously correctly identified tastant concentration is repeated within the same round. To ensure that participants are blinded to the order of tastants, they are not aware of when a round has ended and another round begins.

When two subsequent presentations of a tastant concentration are correctly identified without correct identification of the lower concentration tastant, a tastant sensitivity score is registered for the given tastant. When a tastant is correctly identified, the tastant is replaced with a neutral taste stimulant (pure tap water solvent) in the subsequent semi-randomized rounds until tastant sensitivity scores for all four tastants are registered [9].

### 2.4. Coffee Administration

For consistency in coffee brewing, taste, bitterness, and acidity between participants, we used Nespresso^®^ capsules. This decision was guided by the 2017 Danish champion in black coffee brewing. The choice of the capsule type—*Arpeggio*—was guided by Nespresso, Aarhus, Denmark; partly due to its high popularity among the coffee options in the assortment, as well as balanced characteristics. The *Arpeggio* coffee is a dark roast of Arabica beans from Southern- and Central-America with an intense, yet creamy flavor. It is rated medium-high in intensity (9/12), bitterness (4/5), body (4/5), roast (4/5), and medium in acidity (2/5) [28]. These ratings result from comprehensive sensory evaluation of each type of coffee by Nespresso’s panel of certified cuppers. Furthermore, the *Arpeggio* coffee capsule offers the advantage that it is made in a decaffeinated version with similar flavor ratings on all parameters [29]. The coffee was served as an espresso (40 mL) and a serving temperature of approximately 35–40 °C. All participants were served using the same coffee, cup, temperature, machine, and water source.

### 2.5. Statistical Methods

Statistical analysis was performed using JMP 13.0 (SAS Institute, Cary, NC, USA). Data for both taste tests are presented as means with 95% confidence intervals (CI 95%). Tastant threshold scores were compared (paired *t*-test). MANOVA was used to investigate the effects of daily coffee consumption and PROP insensitivity on the post-coffee-intake modulation of chemosensory sensitivity. Spearman’s *ρ* was used to investigate correlation between taste sensitivity and demographic variables. The criterion for statistical significance was set at alpha = 0.05.

## 3. Results

**Figure 1:**
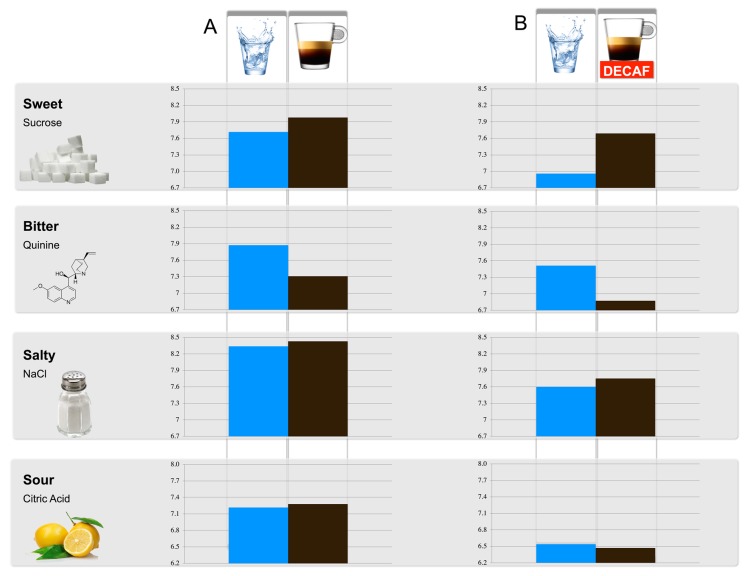
Tastant detection thresholds (dilution steps 1–10). (**A**): testing after water consumption and regular coffee consumption. (**B**): testing after water consumption and decaffeinated coffee consumption (DECAF: decaffeinated coffee).

In the primary study, olfactory and gustatory thresholds were tested before and after coffee consumption. Two minutes after regular coffee consumption, the detection threshold for the sweet tastant was increased while the threshold for the bitter tastant was significantly decreased. These results indicate that participants could detect a lower concentration of sweet, while a higher concentration of bitter was required (Figure 1). The gustatory detection thresholds for the salty and sour tastants did not change significantly. No significant differences were observed between the olfactory threshold measurements after water and regular coffee consumption (*p* = 0.619).

Following the lack of evidence that coffee intake affects olfactory threshold (primary study), in the exploratory study, only gustatory thresholds were tested before and after decaffeinated coffee consumption. Similar changes in the threshold of specific tastants were found for both studies, i.e., sensitivity for bitterness is decreased, sensitivity for sweetness increased, while salty and sour sensitivity remained unchanged (Table 2).

The effect of regular coffee on changes in taste sensitivity was independent of daily coffee consumption for increase in sweet sensitivity (*F*(1,99) = 0.5192, *p* = 0.473). However, the decrease in bitter sensitivity (*F*(1,99) = 4.7975, *p* = 0.031) was found to be larger in participants that did not consume coffee daily (n = 25; decrease in mean bitter sensitivity score of −1.0). Insensitivity to PROP had no effect on sweet sensitivity changes (*F*(1,99) = 0.8353, *p* = 0.363) or bitter sensitivity changes (*F*(1,99) = 0.4587, *p* = 0.500). We found no effects of gender on the changes in sensitivity for sweet (*F*(1,99) = 0.0489, *p* = 0.826) or bitter (*F*(1,99) = 0.3949, *p* = 0.531).

The larger differences in bitter and sweet sensitivity found in the decaffeinated study are likely to reflect the shorter time interval (after coffee consumption) before gustatory testing was performed (See Supplementary Figure S1 for study timeline).

Coffee consumption was slightly positively correlated with age (*ρ* = 0.3381, *p* < 0.001). There was a small negative correlation between baseline tastant sensitivity and coffee consumption for all tastants: sweet (*ρ* = −0.17, *p* = 0.086); bitter (*ρ* = −0.20, *p* = 0.042); salty (*ρ* = −0.08, *p* = 0.420); sour (*ρ* = −0.07, *p* = 0.504).

## 4. Discussion

We identified a change in detection thresholds for sweet taste (increased sensitivity) and bitter taste (decreased sensitivity) in the minutes following coffee consumption. There was no change in olfactory sensitivity or sensitivity to salty and sour tastants. Our results indicate that while the increase in sweet sensitivity is independent of daily coffee consumption, the level of decrease in bitter sensitivity is associated with coffee consumption habits. For participants without regular coffee consumption, the decrease in bitterness sensitivity after coffee consumption was even more pronounced.

A key reflection on these results is related to the possible source of sensory modulation. Although caffeine is highly soluble in lipids, it rapidly crosses the blood–brain barrier both by diffusion and by a saturable transport system [30], these changes in gustatory sensitivity occurred shortly after consumption, which may indicate a mechanism independent of central caffeine modulation. To control for the effects of caffeine, the study on gustatory sensitivity was reproduced using decaffeinated coffee. Here, similar significant threshold changes were found for sweet and bitter tastants. As such, we conclude that our findings are not driven by caffeine. Instead, to a more general attribute of coffee flavor.

Both sweet and bitter taste is registered by the type II cells in the taste buds. Although the sweet receptors (T1Rs) and bitter receptors (T2Rs) may not expressed in the same cell, they do converge on a common intracellular pathway [31]. Furthermore, activity patterns of peripheral taste neurons have been found to vary with stimulus strength [32] As such, the modulation of tastant sensitivity after coffee consumption may very well occur at the peripheral level.

These results add an important contribution to the current understanding of taste function, by providing new strong evidence that this modulatory process may not be as static as previously assumed. In the previous descriptions of a sweet-bitter taste interaction, bitter tastants (quinine and caffeine) were consistently found to inhibit human perception of sweet taste [13,14]. This is in contrast to the current findings of an increased sensitivity to sweet tastant and a not previously described decrease in subsequent bitter sensitivity.

Previous research has indicated associations between taste sensitivity and food consumption behavior [33]. Specifically, increased taste sensitivity was associated with avoidance of coffee in this questionnaire-based study. The current results support this association. Furthermore, it adds a dynamic dimension to coffee consumption habits and tastant sensitivity, as the decrease in bitter sensitivity found to be larger in participants that did not consume coffee regularly. Although the current study does not infer causality of this association, the dynamic differences based on differences in previous exposure could lead to the hypothesis that tastant sensitivity is more closely linked to consummatory habits than previously assumed.

### 4.1. Limitations

The exploratory study reveals that alterations of gustatory sensitivity for bitter and sweet were reproduced. This underlines that the effects were not caused by caffeine, but instead by a more general mechanism. However, it is beyond the scope of the current study to investigate the underlying mechanisms of altered tastant sensitivity. As such, it is yet unknown whether the effects are caused by local changes in type II taste receptor cells (containing both sweet and bitter receptors [31] or by a more central modulation of gustatory sensitivity. The mechanisms responsible for dynamic changes in bitter sensitivity based on coffee consumption are also unclear.

The current results are based on localized testing on the anterior part of the tongue with small amounts of tastant dilutions. Based on the applied methods, it is unknown how the use of a whole-mouth taste test would affect the current findings.

Further research is needed to investigate whether these effects are caused by local taste receptor inhibition and enhancement or if central regulation is at play.

### 4.2. Perspectives

The findings of this study are relevant from both a clinical and gastronomical perspective. Clinically, the rule of thumb that olfactory testing should not be done within an hour of coffee consumption does not seem to have much relevance, as immediate effects on olfactory threshold were found in the present study, thus complementing earlier evidence of no affects after 30 min [25]. However, for gustatory sensitivity, the current findings may influence gustatory perception in e.g., event related or resting-state functional MRI studies. In different hunger states, a greater brain activation in response to sucrose has been described after a pseudo-randomized presentation of gustatory stimuli in the scanner [34]. Dependent on the order of tastant stimulation, subsequent changes in BOLD signal strength may indeed be a mere reflection of changed peripheral sensitivity. As such, this change in sensitivity can be important to consider when setting up the gustatory stimulation paradigm.

From a gastronomic perspective, the current findings of change in specific tastant sensitivity may in fact topple over a well-balanced meal owing to bitter ingredients in the previous course. On the other hand, this study may serve as a scientific foundation to understanding the successful pairing of black coffee and dark chocolate [35].

## Figures and Tables

**Table 1:** Demographic data for the cohorts “Regular coffee”—primary study—and “Decaffeinated coffee”—exploratory study. 95%CI: 95% confidence interval; TDI: threshold, discrimination, and identification.

	Regular Coffee (n = 101)	Decaffeinated Coffee (*n* = 55)
Age (Mean, (95%CI))	25.5 (24.6–26.6)	24.5 (23.6–25.4)
Gender (M/F)	46/55	25/30
Smoker (n,%)	15 (15%)	7 (13%)
Olfactory TDI (Mean, (95%CI))	33.3 (32.6–34.0)	-
Coffee/day (Mean, (95%CI))	2.1 (1.7–2.5)	1.5 (1.1–1.9)

**Table 2:** Mean olfactory and gustatory scores before and after regular/decaffeinated coffee consumption. 95% confidence interval in parenthesis.

	**Sensitivity Score After Water (*n* = 101)**	**Sensitivity Score After Regular Coffee (*n* = 101)**	**Difference** **(*p*-Value)**
Olfactory threshold	6.98 (6.68–7.28)	7.06 (6.72–7.40)	0.08 (*p* = 0.619)
Sweet tastant	7.72 (7.55–7.90)	7.98 (7.84–8.18)	0.26 (*p* < 0.001)
Bitter tastant	7.87 (7.64–8.11)	7.31 (7.08–7.54)	−0.56 (*p* < 0.001)
Salty tastant	8.34 (8.10–8.57)	8.43 (8.19–8.65)	0.09 (*p* = 0.363)
Sour tastant	7.22 (7.05–7.39)	7.28 (7.09–7.46)	0.06 (*p* = 0.379)
	**Sensitivity Score after Water (*n* = 55)**	**Sensitivity Score after Decaffeinated Coffee (*n* = 55)**	**Difference** **(*p*-value)**
Sweet tastant	6.96 (6.70–7.22)	7.69 (7.44–7.95)	0.73 (*p* < 0.001)
Bitter tastant	7.51 (7.12–7.89)	6.87 (6.53–7.22)	−0.64 (*p* < 0.001)
Salty tastant	7.60 (7.26–7.93)	7.75 (7.44–8.05)	0.15 (*p* = 0.306)
Sour tastant	6.54 (6.20–6.89)	6.47 (6.18–6.76)	−0.07 (*p* = 0.598)

## Data Availability

Chemosensory sensitivity data is uploaded according to the transparency policy of the journal.

## Supplementary Materials

The following are available online at https://www.mdpi.com/2304-8158/9/4/493/s1, Figure S1: Study timeline.

## Public Significance Statements

Results of this study revealed that drinking coffee (regular and decaffeinated) subsequently changes our ability to perceive taste, but not smell. After coffee consumption, sensitivity to sweet increase while sensitivity to bitter decrease. This can impact sensory testing procedures, professional tastings, and optimizing food pairings.
