# Supplementary figures and images for: Chemosensory Sensitivity after Coffee Consumption Is Not Static: Short-Term Effects on Gustatory and Olfactory Sensitivity
Source: Foods. 2020 Apr 14;9(4):493. doi: 10.3390/foods9040493 (PMC7230594; doi:10.3390/foods9040493)

## Regular coffee (n=101)

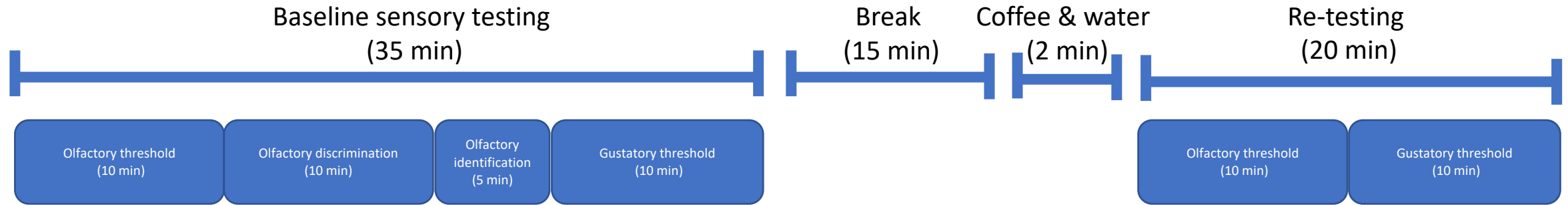

## Decaffeinated coffee (n=55)

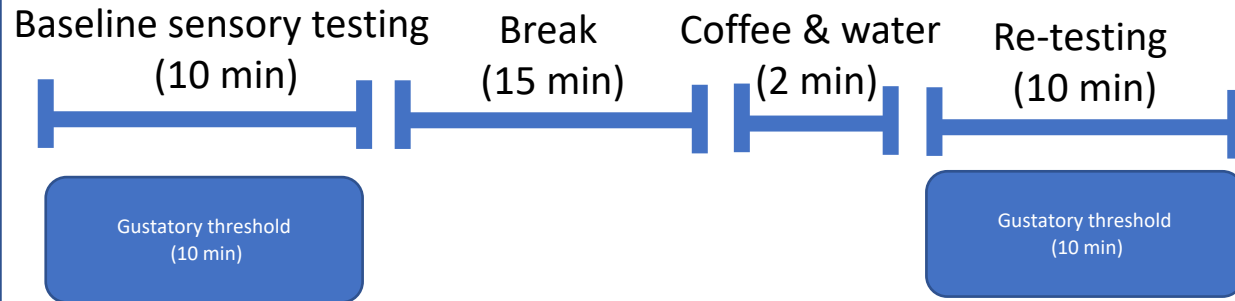

Supplement: Supplementary file 1 [file foods-09-00493-s001.pdf]
